# The State of the Art in Transcriptomics and Proteomics of Clinically Relevant *Sporothrix* Species

**DOI:** 10.3390/jof9080790

**Published:** 2023-07-27

**Authors:** Anna Carolina Procópio-Azevedo, Marcos de Abreu Almeida, Rodrigo Almeida-Paes, Rosely Maria Zancopé-Oliveira, Maria Clara Gutierrez-Galhardo, Priscila Marques de Macedo, Evandro Novaes, Alexandre Melo Bailão, Célia Maria de Almeida Soares, Dayvison Francis Saraiva Freitas

**Affiliations:** 1 Laboratório de Micologia, Instituto Nacional de Infectologia Evandro Chagas, Fundação Oswaldo Cruz, Rio de Janeiro 21040-360, RJ, Brazil; 2 Laboratório de Pesquisa Clínica em Dermatologia Infecciosa, Instituto Nacional de Infectologia Evandro Chagas, Fundação Oswaldo Cruz, Rio de Janeiro 21040-360, RJ, Brazil; 3 Setor de Genética, Departamento de Biologia, Universidade Federal de Lavras, Lavras 37203-202, MG, Brazil; 4 Laboratório de Biologia Molecular, Instituto de Ciências Biológicas, Universidade Federal de Goiás, Goiânia 74690-900, GO, Brazil

**Keywords:** Sporotrichosis, *Sporothrix*, proteome, proteomics, mass spectrometry, transcriptome, transcriptomics

## Abstract

Proteomics provide a robust approach to profile and quantify proteins within cells, organs, or tissues, providing comprehensive insights about the dynamics of cellular processes, modifications, and interactions. Similarly, understanding the transcriptome is essential to decipher functional elements of the genome, unraveling the mechanisms of disease development and the molecular constituents of cells and tissues. Some thermodimorphic fungi of the genus *Sporothrix* cause sporotrichosis, a subcutaneous mycosis of worldwide relevance. The transcriptome and proteome of the main *Sporothrix* species of clinical interest can elucidate the mechanisms underlying pathogenesis and host interactions. Studies of these techniques can contribute to the advancement of novel diagnostic and therapeutic strategies. A literature review was carried out, addressing all articles based on proteomics using mass spectrometry and transcriptomics of *Sporothrix* spp. Twenty-one studies were eligible for this review. The main findings include proteins and genes involved in dimorphism, cell differentiation, thermotolerance, virulence, immune evasion, metabolism, cell adhesion, cell transport, and biosynthesis. With the spread and emergence of sporotrichosis in different countries, ongoing research efforts and new discoveries are welcome to advance knowledge about this mycosis and its agents.

## 1. Introduction

### 1.1. Sporotrichosis

Sporotrichosis is a subcutaneous mycosis caused by thermodimorphic fungi of the genus *Sporothrix*, which are distributed worldwide, especially in tropical and subtropical regions, with areas of high endemicity in Latin America [1,2]. Nowadays, the genus *Sporothrix* encompasses over 50 described species, with 7 species of clinical interest. Those directly involved in clinical cases include *Sporothrix schenckii*, *Sporothrix brasiliensis*, *Sporothrix globosa,* and *Sporothrix luriei*. The species *Sporothrix pallida*, *Sporothrix mexicana,* and *Sporothrix chilensis* can also cause sporotrichosis, but they are more frequently isolated from environmental sources [1,3,4,5,6,7].

The classic form of sporotrichosis transmission occurs through a cutaneous trauma with wooden splinters or rose bush thorns, for example, and affects mainly farmers, gardeners, or individuals who have contact with these and other organic substrates, which are the habitat of the sporotrichosis agents that grow in their filamentous phase in these substrates [8,9,10,11]. Rare cases of infection through the inhalation of fungal conidia may occur, causing pulmonary sporotrichosis [12]. Cat-transmitted sporotrichosis is another form of transmission that has been predominant in Brazil and more recently reported in other countries of South America and abroad. In this case, the disease is transmitted through cat scratching/biting or close contact, affecting mostly housewives, children, older adults, and other people who deal with these animals [1,13,14].

The clinical forms of sporotrichosis are variable and related to the immune status of the host, the burden and depth of the fungal inoculum, and the pathogenicity and thermotolerance of the fungal strain [11,15,16,17]. There are localized forms, with sole involvement of the skin, corresponding to the most part of the clinical presentations, and disseminated forms, which are less frequent, with skin, pulmonary, bone, meningoencephalic, and mucosal involvement [18,19]. Associated hypersensitivity manifestations are frequently observed in zoonotic sporotrichosis [20].

Culture is the reference standard test for the laboratorial diagnosis of sporotrichosis and is based on the fungal isolation from clinical specimens, followed by macro and micromorphological identification and in vitro thermoconversion to the yeast-like phase. The *Sporothrix* spp. structures are rarely visualized on the direct microscopic exam in humans. In cats, direct microscopic examination is more reliable due to the large amount of fungal cells in the tissues, presenting a sensitivity of almost 85%, although culture remains the gold standard test [1,21,22]. The isolation of the fungus can be difficult in some cases, depending on the location of the lesion, as occurs in extracutaneous and atypical disease forms. To overcome this limitation, serological tests, such as the enzyme-linked immunosorbent assay (ELISA), are used for routine presumptive diagnosis and therapeutic monitoring in certain institutions, which presents good clinical correlation. This method can also provide the diagnosis even in immunocompromised patients [23,24,25].

The classification of *Sporothrix* species only using phenotypic methods can be inconclusive, due to intraspecific variations. To provide reliable identification of species, molecular methods are strongly necessary. They are also useful for epidemiological studies involving the different *Sporothrix* pathogenic species, as they differ, for example, depending on the geographic distribution, virulence, and susceptibility to antifungal drugs [1,26].

The treatment of patients with sporotrichosis is chosen according to the clinical form and the immune status of the host. The currently available drugs include saturated potassium iodide solution (SSKI) and the antifungals itraconazole, terbinafine, amphotericin B, and posaconazole. Itraconazole is the drug of choice due to its effectiveness and low risk. [1,27,28,29,30]. Amphotericin B is the drug indicated for the most severe forms of sporotrichosis [1,28,31]. Other therapies can be used alone or as an adjuvant treatment when drugs are contraindicated. These include thermotherapy, cryosurgery with liquid nitrogen, and electrosurgery [1,32,33,34,35,36].

### 1.2. Proteomics

Proteins are responsible for most of the physiological functions of cells, also serving as important pharmacological targets and biomarkers of diseases. Proteomic analysis allows for the identification, quantification, and evaluation of proteins involved in various processes, providing, for example, the discovery of new therapeutic targets, modifications, and interactions with other proteins. Several techniques are applied to identify proteins and quantify them such as electrophoresis, chromatography, mass spectrometry (MS), and bioinformatics [37], and the application of MS-based proteomics in fungal biology, fungal-host interactions, virulence, antifungal development, and therapeutic approaches resulted in major contributions. Proteomic techniques provide a robust approach to profile and quantify proteins within cells, organs, or tissues, generating comprehensive information about the dynamics of cellular processes, modifications, and interactions. They can also identify and characterize biological markers of a given pathogen, helping in the early diagnosis, in the follow-up of the treatment of diseases and in the development of vaccines [38,39].

In general, the methodologies applied in proteomics can be classified as bottom-up, which analyzes peptides through proteolytic digestion, and top-down, which analyzes intact proteins. These two approaches have their own advantages and disadvantages and relative fields of application. The bottom-up type includes chromatographic separation of peptides derived from proteolytic digestion of intact proteins. Intact proteins are digested into peptides prior to analysis via MS, where they are then detected and fragmented. It has the advantage of high sensitivity and easy execution, even for extremely large and complex proteomes such as cell lysates or serum, which can be analyzed with confidence. However, very rarely does this approach covers the entire protein sequence, being limited to 50 to 70% coverage. That is, 50 to 30% of the proteins remain unknown. In addition, a small peptide may be lost during chromatography or a large one may not generate adequate mass spectra. The top-down methodology, on the other hand, is a process in which the proteins remain intact (they do not suffer proteolytic digestion) and are submitted to analysis via MS. This approach allows a better characterization of post-translational modifications compared to peptide-based proteomics. However, larger amounts of samples are required, and the time of analysis is longer, precluding high-throughput analyses. Furthermore, proteins are generally more complicated to handle than small peptides, largely due to their low solubility. Both approaches are associated with several advantages and limitations, and the appropriate approach must be chosen depending on the need [40,41,42,43,44].

### 1.3. Transcriptomics

The term transcriptome was created to refer to the complete set of ribonucleic acids (RNAs), such as messenger RNAs, ribosomal RNAs, transfer RNAs, and microRNAs, in a cell type or tissue, and its analysis allows the identification of these RNAs expressed in an organism or tissue. Therefore, it is a direct reflection of gene expression. Understanding it is essential to interpret functional elements of the genome and the development of diseases as well to reveal the molecular constituents of cells and tissues. Large-scale transcriptional studies have become the best option for the identification of transcriptomes and differential transcripts between cell types and organisms [45,46]. Approaches involving the transcriptome can be used as an alternative to search for information about fungal pathogenicity mechanisms. This occurs through the analysis of gene expression profiles associated with fungal infections, which allows for the identification of potential determinants that facilitate the success of the infection, survival of the fungus in the host, and its virulence, in addition to better understanding fungus–host interaction [47,48,49].

Prior to the development of new sequencing methods, which allowed the development of large-scale RNA sequencing (RNA-Seq), other technologies were developed to quantify the transcriptome. These techniques can be classified in hybridization- or sequence-based approaches. Hybridization-based approaches, in addition to being relatively inexpensive, have a high yield. These typically involve incubating fluorescently labeled cDNA with custom, commercial, or engineered microarrays. However, they have limitations in detection, due to the background and saturation of the signal, making comparisons of the expression levels in different experiments difficult. Hybridization is often laborious and requires complicated normalization methods. Sequence-based approaches allow for the direct determination of the cDNA sequence with the aid of expressed sequence tags (ESTs). However, they have limitations, including an expensive sequencing technology, in addition to the fact that only part of the transcripts is analyzed, compromising the annotation of the transcriptome structure [46,50].

With the creation of affordable next-generation sequencing (NGS) platforms, RNA-Seq has become one of the most important tools for studying the transcriptome. The basic steps of an RNA-Seq experiment include RNA extraction, RNA fragmentation, cDNA generation, the addition of adapters (for the anchoring of sequencing primers and for barcoding and sequencing multiple samples in the same run), the preparation of the library, and sequencing on an NGS platform. Library preparation varies depending on the type of RNA being analyzed, and may differ in fragment size, structural features, abundance, and sequence. After sequencing, the reads can be aligned to a reference genome or assembled de novo. When aligned with a reference genome, it is possible to identify which genes these readings originated from. The assembly of de novo transcripts uses the sequencing reads and is generally performed when there is no reference genome available for the studied species. Independent of the strategy, RNA-Seq can accurately estimate gene expression (at the transcripts level) of all genes, in parallel, under different conditions and allows the discovery of new genes and transcription patterns, helping to understand cell function and metabolic mechanisms. In addition to being highly accurate for quantifying expression levels, it has greater sensitivity and specificity, being able to detect rare and low-expression transcripts [46,50,51,52].

## 2. Methodology

**Figure 1:**
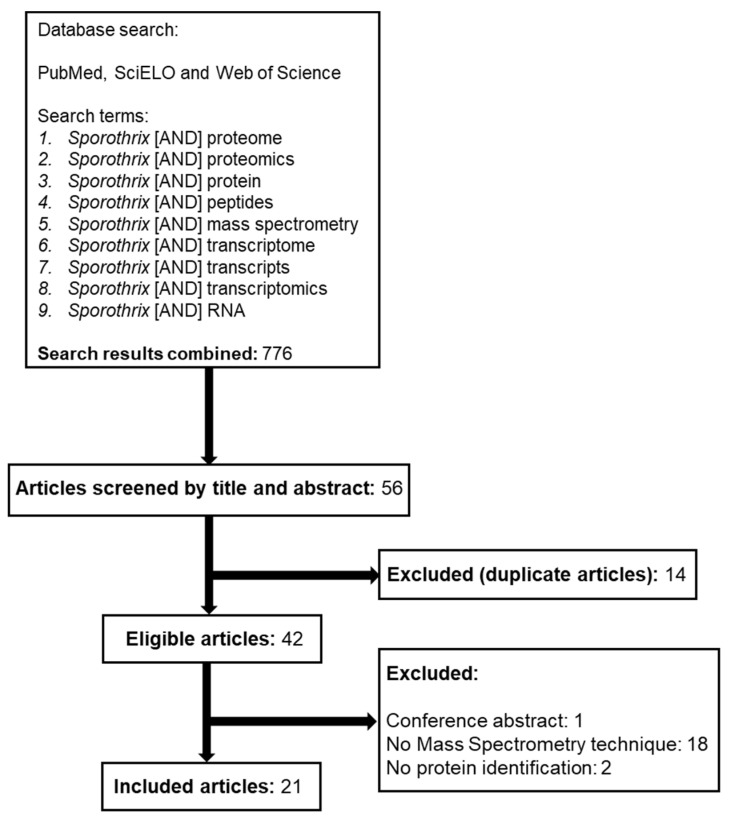
Flowchart detailing the steps of the articles search and those selected to be included in this study.

A literature review was carried out in three different databases: Pubmed/MEDLINE, Scientific Electronic Library Online (SciELO), and Web of Science. The bibliographic survey was conducted up to 23 May 2023, including articles published from 1948 up to May 2023. The review addressed articles on proteomics by means of mass spectrometry and transcriptomics for *Sporothrix* spp. published so far. For this purpose, broader terms were used in the initial search (*Sporothrix* [AND] proteome, *Sporothrix* [AND] proteomics, *Sporothrix* [AND] protein, *Sporothrix* [AND] peptides, *Sporothrix* [AND] mass spectrometry, *Sporothrix* [AND] transcriptome, *Sporothrix* [AND] transcripts, *Sporothrix* [AND] transcriptomics or *Sporothrix* [AND] RNA). The retrieved articles were analyzed in a first round for the selection of those dedicated to the specific topics of interest. Articles for which, based on their title or abstract, it was perceived that they were not describing sporotrichosis/*Sporothrix* spp. studies, did not perform proteomic or transcriptome analysis, were review articles, or contained a search word but did not meet the inclusion criteria were excluded in this first screening. Later, duplicate articles, conference abstracts, and articles with no MS technique or no protein identification were excluded from this review. As a result, only articles published in English were found. The search flow is detailed in Figure 1.

## 3. Results

The search retrieved 21 articles, being 17 dedicated to proteomics (Supplementary Table S1) and 4 to transcriptomics (Supplementary Table S2).

### 3.1. Proteomics Involving Sporothrix spp.

Antibodies with defined specificity have different degrees of protection, like the one mediated by monoclonal antibodies (mAbs) against some pathogenic fungi, modifying the course of the infection in mice. In a study by Nascimento et al. [53], an immunoglobulin G (IgG)1 mAb (P6E7) was produced against the 70-kDa glycoprotein (gp70) of *S. schenckii* to evaluate the effect of passive immunization of mice infected with *S. schenckii* yeast cells. Following a sodium dodecyl sulfate polyacrylamide gel electrophoresis (SDS-PAGE) and gp70 trypsinization, an analysis by means of matrix-assisted laser-desorption/ionization time-of-flight mass spectrometry (MALDI-TOF/MS) allowed the identification of 26 peptides. Gp70 and other molecules secreted by *S. schenckii* to extracellular matrix proteins (ECM) were separated via SDS-PAGE to assess their ability to adhere. Purified gp70 was shown to be recognized by these ECMs in Far-Western blot assays. A significant reduction in the number of CFUs in the spleen and liver of mice was observed when the mAb was injected before and during infection. Thus, it is suggested that passive immunization against gp70 can increase cellular mediation in the immune response and inhibit the adhesion of *S. schenckii* to the host tissues and/or the extra-cellular matrix, aborting the infection. For the first time, it was reported that antibodies play an important role in the host and may or may not nullify the establishment and development of sporotrichosis.

It has already been shown in previous studies, as cited above, that gp70 is an important antigen that is expressed on the fungal cell surface and that it may play a key role in immunomodulation and host response. The objective of the study by Castro et al. [54] was to comparatively evaluate the virulence and expression of gp70 in *S. brasiliensis* clinical isolates and in two reference *S. schenckii* strains. The same mAb used by Nascimento et al. [53] against the gp70 antigen (P6E7) was applied in a Western blot to evaluate the expression of gp70 on the surface of *S. schenckii* and *S. brasiliensis*. A reduced level of gp70 expression was found in all virulent isolates of *S. brasiliensis*, which may represent an imbalance in the immune response, as this antigen induces a protective response against the fungus. A subcutaneous model of murine infection was established to compare virulence and spreadability. The results showed that all but one *S. brasiliensis* isolates had increased pathogenicity compared to *S. schenckii*. For the identification of gp70, cell wall extracts from *S. schenckii* and *S. brasiliensis* were used. Protein bands recognized by mAb P6E7 were manually excised from SDS-PAGE 1D gels, trypsinized and analyzed using MALDI-TOF/MS. The identified peptide sequences matched to cell wall gp70 for both species. Based on the results, this glycoprotein was located in several subcellular sites, including the cell surface, suggesting that gp70 may play a dual role, depending on its location in the fungal cell, and may be associated with adhesion and immunomodulation of the host response. This was the first study to report the identification, expression level, and subcellular location of gp70 from *S. brasiliensis* and *S. schenckii* and its possible correlation with the virulence profile of these two species.

Rodrigues et al. [55] sought to investigate molecules expressed during human sporotrichosis which provoke the humoral response using an immunoproteomic approach through the characterization of IgG-reactive proteins. For the evaluation of the antigenic profile, *Sporothrix* yeast extracts were separated via 2D-PAGE and probed with pooled sera from patients with fixed cutaneous and lymphocutaneous sporotrichosis. A total of 53 spots matching the immunoreactive proteins were cut out from fresh 2D Coomassie-stained gels and were identified via MALDI-TOF/MS. The gp70 was identified in several protein spots as an antigen with homology to 3-carboxymuconate cyclase. The results demonstrated that gp70 was the main cross-linked immunogenic antigen between *S. brasiliensis*, *S. schenckii,* and *S. globosa*. Gp70 showed the best immunoreactivity, while the other proteins, such as F-type H+-transporting ATPase, subunit beta, saccharopepsin, signal peptidase protein, guanine, nucleotide binding protein (G protein), and catalase/peroxidase, were related to signal transduction and pathogenic or metabolic energy processes. Serum samples from patients with sporotrichosis were probed against gp70, and IgG antibodies recognized this antigen, which is supported by the literature results. The results of this study also indicated that *S. mexicana* does not have a gp70 homolog with isolates with clinical relevance, which could be related to its low virulence.

Later, Rodrigues et al. [56] provided data using an efficient protein extraction protocol for rapid and large-scale proteomic analysis using two-dimensional gel electrophoresis, since the optimization of sample preparation and electrophoresis conditions are fundamental steps for the reproducibility of gel-based proteomic assays. The protocol was established and optimized for *Sporothrix* spp., including *S. brasiliensis*, *S. schenckii*, *S. globosa,* and *S. mexicana*. After extraction, protein concentrations were determined by means of the Bradford method and protein extracts were evaluated according to the amount of protein extracted, the diversity of the bands, the integrity of the samples, and the reproducibility of extraction. Approximately 2 μg of protein preparations were resolved using SDS-PAGE, ranging in size from 10 kDa to 270 kDa with clear differences in protein profiles. The extraction protocol was suitable for the study of *Sporothrix* antigenic molecules, generating samples with high protein content without degradation. To assess the complexity of the samples, these were fractionated via 2D electrophoresis. Proteins were successfully precipitated, and proteome maps were recorded, along with a representative image of *S. schenckii* proteins resolved via 2D gel electrophoresis revealing a complex proteomic profile. The protocol used for protein extraction proved to be compatible with immunoblotting, two-dimensional difference gel electrophoresis (2D-DIGE), and MS, demonstrating the potential for discovering new fungal antigens. The data provided are related to the study by Rodrigues et al. [55].

Previously reported studies showed that the 60 kDa and 70 kDa proteins in *S. brasiliensis* and *S. schenckii*, respectively, were associated with virulence profiles and were the main antigenic molecules in murine and human sporotrichosis [55,57]. Due to a lack of information about feline sporotrichosis and the antigenic components involved in the infection, the study by Rodrigues et al. [58] aimed to explore the diversity of molecules expressed by *S. brasiliensis* and *S. schenckii*, which are recognized by IgG in sera from naturally infected cats. After extraction, protein samples were used in the ELISA approach with sera from cats with confirmed sporotrichosis. *S. brasiliensis* and *S. schenckii* protein profiles were analyzed via SDS-PAGE, where the amount of protein was evaluated, as well as band diversity, sample integrity, and extraction reproducibility. Silver staining revealed numerous proteins ranging from 10 kDa to 160 kDa in size, with different intensities. Sera from these cats reacted similarly, with no significant difference in titer between ELISA plates coated with proteins from these two species. To test for antigen diversity, a 1D immunoblot was performed using antigen preparations from four species, where antibodies from cats with sporotrichosis reacted with a wide variety of *S. brasiliensis* and *S. schenckii* proteins. The main molecules recognized by the antibodies present in cat sera were the 60 kDa protein in *S. brasiliensis* and the 70 kDa protein in *S. schenckii*. It has been suggested that this variety of antigenic components may be due to specific antigens secreted by individual fungal strains, as well as to different mechanisms of activation of the immune system of each host. The 3-carboxymuconate cyclase protein (gp60 in *S. brasiliensis* and gp70 in *S. schenckii*) previously identified using MALDI-TOF/MS [55] is the immunodominant molecule in feline sporotrichosis, similar to the disease in mice and humans. Therefore, this molecule may also be useful as a marker in the diagnosis of feline sporotrichosis and as a candidate for vaccine development.

The correlation between molecular data and phenotypic characteristics Is fundamental for the identification of species of the genus *Sporothrix*. And in recent years, researchers have been looking for tools for quick and reliable identification. Thus, Oliveira et al. [59] developed and optimized a new proteomic analysis protocol by means of MALDI-TOF/ MS, or the identification of clinical and environmental isolates of *Sporothrix* spp., based on their proteomic profiles. Analyses were performed with 70 *Sporothrix* spp. isolates, of which 6 reference strains were used to create a reference profile bank for MALDI-TOF/MS identification: *S. brasiliensis*, *S. schenckii*, *S. globosa*, *S. pallida*, *S. mexicana,* and *S. luriei,* and the remaining 64 isolates were used to evaluate this identification flow developed in the study. They were molecularly identified through partial sequencing of the calmodulin gene (*CAL*). For validation, the 64 isolates were also analyzed in MALDI-TOF/MS, demonstrating a similarity of 98 to 99%, allowing the identification at the species level. The results of this study emphasize that MALDI-TOF/MS is a reliable, fast, and accurate method for the identification of *Sporothrix* spp., reducing the time required for its identification, contributing to the diagnosis in mycology laboratories.

Subsequently, a clinical case report of ocular sporotrichosis was made in a patient, with an isolation of *Sporothrix* spp. in a culture of conjunctival secretion. The isolate was subsequently identified at the species level via MALDI-TOF/MS, following the analysis protocols previously described [59]. The acquired spectra were compared in an internal database containing spectral data of species of the genus *Sporothrix*, and the spectrum of the isolate under study showed a MS profile compatible with *S. brasiliensis*. Through using these tools, it was possible to perform a reliable fungal identification at the species level, demonstrating that the MALDI-TOF/MS technique can be an alternative to other identification methods, such as the partial sequence of genes, which require more time for execution and identification [60].

Zhang et al. [61] used a new approach to study dimorphism in *S. schenckii*, helping to better understand the molecular mechanisms of mycelium–yeast transition. The characterization of the *S. schenckii* proteome could be useful for investigations related to those proteins with differential expressions that occur during this transition. Two-dimensional polyacrylamide gel electrophoresis (2D-PAGE) was performed on proteins extracted from both the mycelial and yeast phases. Both gels were analyzed, comparing the expression levels of certain proteins according to spot intensity. The differential expressed proteins were then analyzed via MALDI-TOF/MS, and then identified through using available databases for homologous sequences in other fungi. Twenty-four proteins preferentially expressed in the yeast phase were identified and classified into broad functional categories related to some important functions, such as metabolism, signaling, DNA repair, transport, and stress response, among others. Among these 24 proteins, attention was focused on the histidine protein kinase (HPK) DRK1 and phosphatidylinositol 3-kinase (PI 3-kinase), two enzymes related to signal transduction. It was inferred that DRK1 could act as a sensor of environmental change in *S. schenckii*; however, its pathogenic role would be still under investigation. Differential expression in PI 3-kinase in the yeast phase could serve to resist temperature and osmotic stresses and to help *S. schenckii* to survive in vivo. Proteomic methodologies associated with the *S. schenckii* morphological transition may help to understand the molecular events associated with this fundamental phenomenon of fungal pathogenesis.

In addition to dimorphism and thermotolerance, fungal proteins linked to virulence factors and immune system evasion deserve attention. One study compared the genome and the proteome expressed in *S. brasiliensis* and *S. schenckii* yeast cells to assess why *S. brasiliensis* is more virulent and pathogenic. Bottom-up ultra-high-resolution MS analyses allowed the identification of proteins involved in immune evasion, metabolism, adhesion, and cell surface and biosynthetic processes, with 60 proteins expressed exclusively by *S. brasiliensis*. Through a comparative analysis with existing literature data, the authors identified nine proteins: extracellular cell wall glucanase, aminopeptidase I, Mn superoxide dismutase, heat shock 70-kDa protein 1/8, glyceraldehyde-3-phosphate dehydrogenase (GAPDH), hydroxymethylglutaryl-coenzyme A (HMG-CoA) lyase, progesterone binding protein, rhamnolipid biosynthesis 3-oxoacyl-(acyl-carrier-protein) reductase, and acetyl-CoA hydrolase. They were considered potentially involved in fungal virulence and immune evasion in other species, many of which had not yet been reported for the analyzed *Sporothrix* species. These proteins have functions related, for example, to the evasion of the immune system; the secretion of toxins; the metabolism of lipids, proteins, and carbohydrates; and the production of biofilm; among others. This study was the first to characterize and compare the proteomes of *S. brasiliensis* and *S. schenckii*, opening space for new studies aimed at validating new virulence markers in *S. brasiliensis* [62].

De Almeida et al. [63] proposed an immunoproteomic approach evaluating antigenic proteins from an *S. brasiliensis* strain isolated from an infected cat, for the development of an effective vaccine against sporotrichosis. By means of two-dimensional Western blot analysis, it was possible to observe 53 antigenic spots in the *S. brasiliensis* proteome with the serum of infected mice. Among them, 16 were selected for further identification of seroreactive proteins via MALDI–TOF/MS. Of those 16 spots, thirty-four immunogenic proteins from 14 spots were identified, and two could not be identified. These were related to virulence, metabolic activities, or unknown functions. Of these 34 proteins, three peptides, ZR3 (importin), ZR4 (hypothetical protein), and ZR8 (GP70), were selected and induced a protective immune response against sporotrichosis. A vaccine model with these peptides was developed in mice, and the results indicated that the diameter of the lesions was reduced. Peptides ZR8 and ZR3 demonstrated a better protective immune response mediated by CD4 + T cells and a good prognosis, being considered possible vaccine targets for the treatment of sporotrichosis.

Extracellular vesicles (EVs) are released by some fungi and can interact with the host cell and modulate the host’s immune response. They are associated with the transport of molecules across the cell wall along with RNA, lipids, polysaccharides, proteins, and other components. These immunogenic components contribute to drug resistance, facilitating invasion and pathogenesis. The objective of this study was to analyze part of the composition of EVs from *S. brasiliensis* yeast cells, its potential to modulate dendritic cells (DCs), and promote protection in an experimental sporotrichosis model. To analyze the effect of *S. brasiliensis* EVs during in vivo infection, three groups of 15 animals received two doses of EVs at different concentrations. The lesions were characteristic of sporotrichosis in all infected animals. However, in animals that previously received EVs, a larger mean diameter of the lesions and a greater fungal load were observed, demonstrating that the greater the number of inoculated EVs, the greater the degree of fungal growth, suggesting that EVs favor the pathogenesis of the fungus on the skin. Liquid chromatography coupled to mass spectrometry (LC-MS/MS) was performed to verify the protein composition of the EVs, and 63 proteins were found in *S. brasiliensis*, 40 proteins in *S. schenckii*, and 4 proteins in both species. In the proteomic analysis, the identified proteins were related to several processes, such as metabolism and transport, in addition to acting as virulence factors. Serine/threonine protein kinases were also found, which also play a role in the establishment of the pathogen. This study suggests that *S. brasiliensis* EVs play an important role in fungal virulence and can interact with the host’s immune system, favoring its establishment [64].

A study by Ruiz-Baca et al. [65] aimed to identify proteins potentially involved and differentially expressed in the response to oxidative stress after exposure of *S. schenckii* yeast cells to increasing concentrations of the oxidative agent, hydrogen peroxide. These proteins were analyzed using 2D-PAGE and identified using MALDI–TOF/MS. At least five differentially expressed proteins were identified, like a 70 kDa heat shock protein (Hsp70), GroEL chaperonin, elongation factor 1- (EF-1), mitochondrial peroxiredoxin (Prx1) and a hypothetical protein. Hsp70 was suggested as an important protein in the interaction with host cells and in the response to oxidative stress. Studies indicate overexpression of GroEL chaperonin under cellular stress conditions. EF-1 has a role in the stress response, as a modulator of the transcription of other proteins, and mitochondrial Prx1 which are enzymes that protect cells against oxidative stress. With these findings, it was proposed that these proteins are possibly involved in the resistance of the fungus to oxidative stress, allowing *S. schenckii* to evade host phagocytes and succeed in the infection.

In a study by Félix-Contreras et al. [66], a proteomic analysis of the cell wall of *S. schenckii* was performed in response to the oxidative agent menadione, since pathogenic fungi can survive inside phagocytes during oxidative stress. This capacity is obtained via mechanisms that depend, at least partially, on the cell wall. However, the role of cell wall proteins during these oxidizing conditions is not completely understood. After obtaining the cell wall proteins, their identification, in control samples and under oxidative stress, was performed via liquid chromatography electrospray ionization tandem mass spectrometric (LC-ESI-HDMSE) analysis. About 76 proteins were differentially expressed between control cells and cells exposed to menadione. Based on bibliographical research on these 76 proteins, 13 were identified as potentially involved in the mechanisms of oxidative stress response and that could be in the cell wall of the fungus. The proteins identified were thioredoxin1 (Trx1), superoxide dismutase (Sod), GPI-anchored cell wall protein, β-1,3-endoglucanase EglC, glycoside hydrolase (Gh), chitinase, CFEM domain protein, glycosidase crf1, covalently linked cell wall protein (Ccw), 30 kDa heat shock protein (Hsp30), lipase, trehalase (Treh), fructose-bisphosphate aldolase (Fba1), and citrate synthase (Cs). They would be involved in protection against oxidative stress, fungal virulence and reproduction, nutrient acquisition, adhesion to host cells, and evasion of the immune response through cell wall restructuring. The results indicated that *S. schenckii* has different enzymatic mechanisms that allow the detoxification of reactive oxygen species mediated by phagocytes of the human host, allowing for its evasion of the immune system and accentuating the infection.

Subsequently, a study by Saucedo-Campa et al. [67] also aimed to evaluate which cell wall proteins of *S. schenckii* modify their expression in response to another oxidative agent, hydrogen peroxide. The extracted proteins, exposed to this chemical oxidant or not, were analyzed via high-sensitivity ion mobility spectrometry/mass spectrometry using electrodynamic ion funnel interfaces (LC-ESI-IMS-QTof). Twenty-eight proteins associated with the cell wall of *S. schenckii* with differential expression to hydrogen peroxide could potentially be involved in the response to reactive oxygen species and confer protection to this fungus in host phagocytes. Major functional groups of proteins that changed their expression in response to hydrogen peroxide include proteins associated with cellular metabolism, energy production, protein synthesis, and stress response functions. Additionally, it was observed through gene expression analysis that *S. schenckii* is also able to respond at transcriptional and translational levels to escape harmful substances such as hydrogen peroxide even at low concentrations, thus demonstrating that this substance influences the transcription and translation of cell wall proteins that provide protection against oxidative stress. These results could complement the previous study carried out by Ruiz-Baca et al. [65] and Félix-Contreras et al. [66].

A study by Silva-Bailão et al. [68] aimed to provide a more robust proteomic analysis of three human pathogenic species of the genus *Sporothrix*: *S. brasiliensis*, *S. schenckii,* and *S. globosa*. Protein extracts were analyzed using nanoscale liquid chromatography coupled with a tandem MS approach (NanoUPLC-MSE). Enzymes related to lipid metabolism with differential expression among the three species were downregulated in *S. globosa*. In addition to the preferential metabolism of amino acids in *S. brasiliensis*, the overexpression of enzymes involved in lipid metabolism was observed, showing that the yeast phase of this species, in parasitism, is highly adapted for the use of available nutrients. This fact could explain the efficiency of *S. brasiliensis* in the infection; compared to *S. globosa*, *S. brasiliensis* was the only species to express urease, which has an impact on virulence in other pathogenic fungi. The overexpression by *S. brasiliensis* of enzymes involved in the hydrolysis of glycosidic bonds, which act in resistance to osmotic stress, was also observed. These results suggest that cell wall remodeling may be more pronounced in *S. brasiliensis*. This remodeling can be a relevant aspect faced in its environmental adaptation in relation to virulence. Thus, it was verified that *S. brasiliensis* has a differentiated metabolism compared to other species, mainly in relation to amino acids and cell wall remodeling. These findings may explain its more virulent nature and ability to infect a wide range of mammals. This study was the first report of a comparative analysis of the protein level between the three main species causing sporotrichosis.

The binding of soluble pattern recognition proteins (sPRPs) on the surface of the pathogen can lead to different cellular responses related to its elimination through specific receptors on macrophages. To identify human serum proteins with potential involvement in humoral immunity, Beltrán et al. [69] analyzed human serum proteins that bind to *S. schenckii* conidia and increase phagocytosis. Using LC/ESI–MS/MS analysis, the isolation of plasma proteins capable of binding to conidia revealed at least four proteins identified as albumin, transferrin, serum amyloid component (SAP), and α-1 antitrypsin (AAT). Assays involving conidial phagocytosis demonstrated that SAP and AAT moderately increased pathogen internalization, while transferrin and albumin did not. Thus, experiments involving the immune response to *S. schenckii* coated with SAP and AAT may help in understanding this interaction in the innate immune response.

The cell wall of *S. schenckii* has a component called peptidorhamnomannan (PRM), which gives the fungus immunogenicity and specific epitopes. Although it is well studied, not much is known about the identities of the proteins that make up this glycoconjugate. An LC-MS/MS analysis was performed to obtain more information about those proteins and evaluate their contribution to the *S. schenckii*–host interaction. Of the PRMs found, two stood out for their high coverage, namely, heat shock protein 60 (Hsp60) and Pap1. Hsp60 is a highly conserved protein and was found in *S. brasiliensis*, *S. schenckii,* and *S. globosa*, while Pap1 was found only in *S. schenckii* and *S. brasiliensis*, suggesting a correlation between virulence and the presence of this protein in the fungus during *S. schenckii*–host interaction. Recombinant proteins and polyclonal anti-rHsp60 and anti-rPap1 antibodies were used in ELISA-based experiments to evaluate *S. schenckii* adhesion to extracellular matrix proteins. A blockade of Hsp60 and Pap1 was performed with these polyclonal antibodies, which indicated that they are important agents in the *S. schenckii*–host interaction and suggesting that they contribute to its adhesion, with distinct adhesion properties. The role of Hsp60 and Pap1 in *S. schenckii* virulence was evaluated in *Galleria mellonella* larvae. When *S. schenckii* cells were inoculated into *G. mellonella* larvae, both rHsp60 and rPap1 conferred protection against experimental infection, suggesting that they are adhesins that make an important contribution to the virulence of this species [70].

### 3.2. Transcriptomics Involving Sporothrix spp.

A study by Giosa et al. [71], taking comparative transcriptomic analyses of the mycelium and yeast phases, sought to unearth the molecular mechanisms relying on *S. schenckii* dimorphism. A new annotation of the genome of *S. schenckii* from its RNA-Seq data, available in the *Sporothrix* Genome DataBase, was also presented, since a significant fraction (>30%) of the reads made for the reference genome would be in unannotated genomic regions. The RNA of the two phases was extracted, then sequenced on the Illumina NextSeq 500 platform, and, before gene expression analysis, the existing genome of *S. schenckii* was annotated. According to the new genome annotation, 98.9% of the previously reported protein-coding genes were present. In addition, 132 new protein-coding sequences were identified in genomic regions that were not in the first annotation version [72]. A variation of 87.75% was observed in terms of gene expression due to dimorphism. About 12,911 and 12,104 genes were expressed in the mycelium and yeast phase, respectively, with 10,443 genes expressed in both phases while 2468 and 1661 were mycelium- and yeast-specific genes, respectively. A total of 8795 genes were differentially regulated between the yeast and mycelium stages of *S. schenckii*, with 4494 genes upregulated in the yeast stage. Upregulation of genes involved in nitrate uptake and arginine biosynthesis, which plays a role in the regulation of morphogenesis and reproduction, has been reported. It was also found that expression of the carbamoyl phosphate synthase gene was downregulated in the mycelium phase compared to that in yeast, suggesting a possible involvement of amino acid biosynthetic pathways in the mycelium–yeast transition. The transcriptomic data reported in this study can serve as a reference for the investigation of genes involved in the dimorphic transition of *Sporothrix* spp. and other biological processes.

He et al. [73] performed a transcriptomic analysis on an *S. schenckii* strain isolated from a patient with disseminated pulmonary sporotrichosis. They focused on genes involved in stress adaptation, aiming to obtain more genetic information related to the dimorphic transition. After RNA extraction, a comparison of gene expression in the mycelium and yeast phases was performed. Illumina sequencing was performed, generating approximately 35.6 million high-quality reads. About 1259 genes showed differential expression in the yeast phase, compared to the mycelium phase, in which 830 were upregulated in yeast phase. These would be strongly associated with adaptation to stress, growth and development, adhesion, and colonization. The expression of other stress-related proteins, such as catalase, oxidoreductase, high osmolarity signaling protein, and pH response regulatory proteins, were also upregulated in the yeast stage. Overexpression of *DRK1*, a protein required for pathogenesis, has been found to be involved in regulating the mycelium-to-yeast transition. The analysis also showed that genes coding for some proteins that may contribute to the transition, such as ATPase, glucanase, glucosidase, lactose regulatory protein, and glucose transporter, were positively regulated in yeast phase. The overexpression of serine proteases, aspartic proteases, aspartic-type endopeptidase, and metalloproteinases was also discovered, which would be related to increased host adhesion and colonization. This study allowed the discovery of information regarding the mechanisms of dimorphism and pathogenesis of *S. schenckii*, which may be useful for similar genetic or genomic studies of other dimorphic fungi.

A STE20-like serine/threonine-protein kinase (SsSte20) was previously identified using two-dimensional electrophoresis, which was overexpressed in the yeast phase, in comparison to the mycelial phase of *S. schenckii*, suggesting that this was part of the morphogenesis and pathogenesis of this fungus [61,74]. With these findings, Hou et al. [75] proposed to investigate the role of SsSte20 in temperature changes and in the dimorphic transition of *S. schenckii* and its target genes, using double-stranded RNA interference (dsRNAi). A comparative analysis of the transcriptome of the standard strain of *S. schenckii* and the RNA interference mutant SsSte20 (SsSte20-i) was performed using the RNA-Seq method to elucidate possible gene correlations with dimorphism and virulence. Total RNA from the standard and SsSte20-i strains was extracted for Illumina sequencing. Sequences were mapped to the reference genome of *S. schenckii*. At 37 °C, for the standard strain, about 117 genes were upregulated and 130 genes were downregulated compared to the SsSte20-i strain. These genes were involved in metabolic pathway processes. Transport proteins and ABC (ATP-binding cassette) transporters were downregulated in the SsSte20-i strain, demonstrating that meiosis and metabolism processes were affected by SsSte20 inactivation. The transcriptome analysis indicated that genes encoding glucan 1,3-beta-glucosidase and methylsterol monooxygenase, which are involved in lipid transport and cell wall remodeling, were affected by SsSte20 silencing. Membrane-associated transporters such as the lysine protein permease LysP, amino acid permease, MFS proteins, and ABC transporters were also significantly affected by the inhibition of SsSte20. These findings demonstrate the role of SsSte20 in the virulence and dimorphic transition of *S. schenckii* and provide a molecular basis for future studies and the development of strategies involving SsSte20 and its related genes.

Another study from this same group also focused on the dimorphic transition. The overexpression of *SsDRK1* and *SsSte20* in the yeast phase signaling pathways compared to the mycelium phase of *S. schenckii* had already been demonstrated [74]. However, due to the lack of more details regarding the signaling pathways that control the dimorphic transition in *S. schenckii*, Zheng et al. [76] performed a transcriptomic analysis of the yeast and mycelium stages to obtain more information about these pathways, including the two-component and heterotrimeric G protein signaling systems, Ras and MAPK cascades. RNA from both phases was extracted and sequenced on the Illumina HiSeq™ Xten platform. About 12,217 genes were differentially expressed between the two phases, with 7271 being upregulated and 4946 being downregulated in the yeast phase. There were significant changes in relation to metabolic processes, oxidation–reduction reactions, and transmembrane transport. Several genes, including *DRK1, Hog1, Skn7*, and *Ste11*, have been altered and are involved in the heterotrimeric G-protein two-component system, cAMP, and Ras-Hog1 signaling pathways, suggesting that these signal transduction pathways play important roles in the dimorphic transition. Several genes in the Ras signaling pathway were significantly upregulated in the yeast phase, suggesting that signal transmission through these heterotrimeric G-protein genes occurs during the early transitional stage. Thus, the analysis of the transcriptome was important for a better understanding of the role of signaling pathways in the mycelium–yeast transition, in addition to providing a basis for further research on the molecular mechanisms related to the dimorphic transition of *S. schenckii*.

## 4. Conclusions

Despite the limited number of transcriptome and proteome studies for *Sporothrix* spp., these, in addition to gaining more visibility nowadays, have proved to be extremely important, as they have enabled the identification and better understanding of the molecular aspects driving its biology. For example, the role of the humoral response to *Sporothrix* spp. is not yet well understood. The fungal cell wall and its components play an important role in host–pathogen interaction. Differences in the chemical composition of the cell wall and the expression of unique proteins are characteristics that can influence evasion and success in infection, contributing to a better understanding of the virulence of species. There are still some challenges related to the identification of antigens that trigger a protective immunoresponse. Therefore, the identification of these molecules constitutes an important step for studies involving possible vaccines against *Sporothrix* spp. For the diagnosis, proteomic characterization of the different species is an effective and reliable alternative in well-equipped laboratories of mycology, as it requires less execution and identification time compared to other molecular methods, such as partial gene sequencing. Transcriptional analyses of *Sporothrix* spp. allow for a better understanding of the molecular mechanisms related to their mycelium–yeast transition. Thus, the study of the transcriptome combined with proteomics serves as a basis for the identification of differentially expressed genes and which proteins are involved in the metabolic processes of *Sporothrix* spp., elucidating antigenic factors, fungal pathogenesis mechanisms, and its interaction with the host. In addition to the peculiarities of each host and its immune system, it is possible that there are correlations between these fungal factors and the different clinical presentations.

## Data Availability

This was a review of already published scientific papers that can be found in the reference list.

## Supplementary Materials

The following are available online at https://www.mdpi.com/article/10.3390/jof9080790/s1, Table S1. Proteins analyzed/found and objectives of the articles on the proteomics of *Sporothrix* spp. Table S2. Gene expression and objectives of the articles on the transcriptomics of *Sporothrix* spp.
