# Supplementary material for: The State of the Art in Transcriptomics and Proteomics of Clinically Relevant Sporothrix Species
Source: J Fungi (Basel). 2023 Jul 27;9(8):790. doi: 10.3390/jof9080790 (PMC10455387; doi:10.3390/jof9080790)
Supplement: Supplementary file 1 [file jof-09-00790-s001.zip › jof-2503091-supplementary.pdf]

Table S1. Proteins analyzed/found and objectives of the articles on the proteomics of *Sporothrix* spp.

**Ref.**	**Proteins found/analyzed**	**Main objective**
[53]	Gp70	Evaluation of the effect of passive immunization of mice infected with *S. schenckii* yeast cells
[54]	Gp70	Virulence and expression of Gp70 in *S. brasiliensis* clinical isolates and *S. schenckii* strains
[55]	Gp70	Evaluation of antigenic profiles of *Sporothrix* species during human sporotrichosis
[56]	--	Protein extraction protocol for rapid and large-scale proteomic analysis
[58]	3-carboxymuconate cyclase protein	Diversity of molecules expressed by *S. brasiliensis* and *S. schenckii* and which are recognized by IgG in serum
[59]	--	Development and optimization of a new proteomic analysis protocol by MALDI-TOF/ MS for the identification of *Sporothrix* spp.
[60]	--	Reliability of fungal identification at the species level, by MALDI-TOF/MS technique
[61]	Histidine protein kinase (HPK) DRK1 and phosphatidylinositol 3-kinase (PI 3-kinase)	Understanding of molecular mechanisms of mycelium-yeast transition in *S. schenckii*
[62]	Extracellular cell wall glucanase, aminopeptidase I, Mn superoxide dismutase, heat shock 70-kDa protein 1/8, glyceraldehyde-3-phosphate dehydrogenase (GAPDH), hydroxymethylglutaryl-coenzyme A (HMG-CoA) lyase, progesterone binding protein, rhamnolipid biosynthesis 3-oxoacyl-(acyl-carrier-protein) reductase, and acetyl-CoA hydrolase	Comparison of genomes and proteomes expressed in *S. brasiliensis* and *S. schenckii* yeast cells
[63]	ZR3 (importin), ZR4 (hypothetical protein) and ZR8 (GP70)	Evaluation of antigenic proteins from a *S. brasiliensis* strain for the development of an effective vaccine
[64]	Serine/threonine protein kinases	Analysis of part of the composition of EVs from *S. brasiliensis* yeast cells
[65]	70 kDa heat shock protein (Hsp70), GroEL chaperonin, elongation factor 1- (EF-1), mitochondrial peroxiredoxin (Prx1) and a hypothetical protein	Identification of proteins potentially involved and differentially expressed in the response to oxidative stress involving hydrogen peroxide
[66]	Thioredoxin1 (Trx1), superoxide dismutase (Sod), GPI-anchored cell wall protein, β-1,3-endoglucanase EglC, glycoside hydrolase (Gh), chitinase, CFEM domain protein, glycosidase crf1, covalently-linked cell wall protein (Ccw), 30 kDa heat shock protein (Hsp30), lipase, trehalase (Treh), fructose-bisphosphate aldolase (Fba1) and citrate synthase (Cs)	Proteomic analysis of the cell wall of *S. schenckii* in response to the oxidative agent menadione
	Peroxiredoxin (Prx), Superoxide dismutase (Sod) (Cu-Zn),	

[67]	GPI-anchored cell wall β-1,3- endoglucanase EglC β-glucosidase, Covalently-linked cell wall protein, β-1,3-glucanosyltransferase,Trehalose synthase (TreS), Heat shock protein 70-5 (Hsp70-5), Glycoside hydrolase, GPI anchored cell wall protein, Lipase 1 (Lip1), Glycosidase crf1, Elongation factor 1-beta (EF-1β), Trehalose-6-phosphate synthase (Tps1), Citrate synthase (Cs), Glyceraldehyde-3-phosphate dehydrogenase (Gapdh), Enolase (Eno), Elongation factor 1-alpha (EF-1α), Phosphoglycerate kinase (PgK), Triosephosphate isomerase (Tpi), Fructose-bisphosphate aldolase (Fba), Pyruvate kinase (Pk), Alcohol dehydrogenase (Adh), Trehalase (Trh), Thioredoxin 1 (Thx) and CFEM domain-containing protein	Evaluation of which cell wall proteins of *S. schenckii* modify their expression in response to oxidative agent, hydrogen peroxide.
[68]	Glycolytic pathway HK (hexokinase), GPI (glucose-6-phosphate isomerase), FBA (fructose biphosphate aldolase), TPI (triosephosphate isomerase) GAPDH (glyceraldehyde 3 phosphate dehydrogenase), PGK (phosphoglycerate kinase), ENO (enolase), PK (pyruvate kinase); TCA cycle PDH (pyruvate dehydrogenase), CS (citrate synthase), ACO (aconitase), OGDHE1 (2-oxoglutarate dehydrogenase E1 component), OGDHE2 (2-oxoglutarate dehydrogenase E2 component), FH (fumarate hydratase), MDH (malate dehydrogenase); vitamin biosynthesis THI4 (thiamine biosynthetic enzyme); fermentation ADH (alcohol dehydrogenase); respiratory chain NDUFAB1 (NADH dehydrogenase ubiquinone 1 alpha beta subcomplex 1); pentose phosphate pathway PGD (6-phosphogluconate dehydrogenase), TKL (transketolase), TAL (transaldolase); amino acid metabolism BCAT (branched-chain amino acid aminotransferase), ASA (aspartatesemialdehyde dehydrogenase), ABAT (4-aminobutyrate aminotransferase), MCC (3-methylcrotonyl-CoA carboxylase beta subunit), ALT (alanine transaminase), HPPD (4-hydroxyphenylpyruvate dioxygenase), CBS (cystathionine beta synthase), CTH (cystathionine gamma lyase); cell wall remodeling GLU (Endo-1,3(4)-glucanase), BGL (beta-glucosidase), TRES (trehalose synthase), UAP (UDP-N-acetylglucosamine pyrophosphorylase); oxidative stress response SOD (superoxide dismutase), CATP (catalase/peroxidase), CAT (catalase), PRX1 (peroxiredoxin), GSR (glutathione reductase), GST (glutathione S transferase); heat shock response GroEL (chaperonin GroEL), GroES (chaperonin GroES), HSP70-4 (heat shock 70 kDa protein 4), HSP70-5 (heat shock 70 kDa protein 5), HtpG (molecular chaperone HtpG), HSP70-18 (heat shock 70 kDa protein 18)	Robust proteomic analysis of three human pathogenic species of the genus *Sporothrix*: *S. brasiliensis*, *S. schenckii* and *S. globosa*.
[69]	Albumin, transferrin, serum amyloid component (SAP) and α -1 antitrypsin (AAT)	Identification of human serum proteins that bind to *S. schenckii* conidia and increase phagocytosis
[70]	Heat shock protein 60 (Hsp60) and Pap1	Evaluation of proteins that compose PRM complex cell wall and evaluate their contribution to the *S. schenckii*-host

		interaction

Table S2. Gene expression and objectives of the articles on the transcriptomics of *Sporothrix* spp.

Ref.	Gene expression	Main objective
[71]	Genes coding proteins involved in the regulation of morphogenesis and reproduction: nitrate uptake and arginine; Biosynthesis and expression of the carbamoyl phosphate synthase gene possible involved of amino acid biosynthetic pathways	*S. schenckii* changes in gene expression profiles associated with dimorphism through transcriptomic analyses of the mycelium and yeast phases
[73]	Expression of stress-related proteins: catalase, oxidoreductase, high osmolarity signaling protein and pH response regulatory proteins. DRK1 expression involved in regulating the mycelium-to-yeast transition; Genes coding for some proteins related to the transition: ATPase, glucanase, glucosidase, lactose regulatory protein and glucose transporter; Genes coding for proteins related to increased host adhesion and colonization: serine proteases, aspartic proteases, aspartic-type endopeptidase, and metalloproteinases	Genes involved in stress adaptation, aiming to obtain more genetic information related to the dimorphic transition in *S. schenckii*
[75]	Genes coding proteins involved in metabolic pathway processes: Transport proteins and ABC (ATP-binding cassette) transporters; Genes coding proteins involved in lipid transport and metabolism of the cell wall: glucan 1,3-beta-glucosidase, and methylsterol monooxygenase, lysine protein permease LysP, amino acid permease, MFS proteins and ABC transporters	Elucidation of possible SsSte20 gene correlations with dimorphism and virulence in *S. schenckii*
[76]	Genes involved in signal transduction pathways: DRK1, Hog1, Skn7 and Ste11	Transcriptomic analysis of yeast and mycelium stages in *S. schenckii*, aiming for more details about the signaling pathways that control the dimorphic transition
